# Association between Polymorphism rs61876744 in *PNPLA2* Gene and Keratoconus in a Saudi Cohort

**DOI:** 10.3390/genes14122108

**Published:** 2023-11-21

**Authors:** Altaf A. Kondkar, Taif A. Azad, Tahira Sultan, Tanvir Khatlani, Abdulaziz A. Alshehri, Glenn P. Lobo, Hatem Kalantan, Saleh A. Al-Obeidan, Abdulrahman M. Al-Muammar

**Affiliations:** 1 Department of Ophthalmology, College of Medicine, King Saud University, Riyadh 11411, Saudi Arabia; mtanwar@ksu.edu.sa (T.A.A.); tasayed@ksu.edu.sa (T.S.); kalantan@ksu.edu.sa (H.K.); alobeidan@yahoo.com (S.A.A.-O.); aalmummar@ksu.edu.sa (A.M.A.-M.); 2 Glaucoma Research Chair in Ophthalmology, College of Medicine, King Saud University, Riyadh 11411, Saudi Arabia; 3 King Saud University Medical City, King Saud University, Riyadh 11411, Saudi Arabia; 4 Department of Blood and Cancer Research, King Abdullah International Medical Research Center, King Saud Bin Abdulaziz University of Health Sciences, Ministry of National Guard Health Affairs, Riyadh 11426, Saudi Arabia; khatlanita@mngha.med.sa; 5 Department of Ophthalmology, Imam Abdulrahman Alfaisal Hospital, Riyadh 14723, Saudi Arabia; abaalshehri@moh.gov.sa; 6 Department of Ophthalmology and Visual Neurosciences, University of Minnesota, Minneapolis, MN 55347, USA; lobo0023@umn.edu

**Keywords:** *APOE*, *CSNK1E*, genetics, genotyping, ophthalmology, *PNPLA2*, rs61876744, rs138380, rs429358, rs7412

## Abstract

The genetic etiology of Keratoconus (KC) in Middle Eastern Arabs of Saudi origin is still unclear. A recent genome-wide study identified two significant loci in the region of *PNPLA2* (rs61876744) and *CSNK1E* (rs138380) for KC that may be associated with KC in the Saudi population. In addition, polymorphisms in the apolipoprotein E (*APOE*) gene, namely, rs429358 and rs7412, responsible for *APOE* allelic variants ε2, ε3, and ε4, may influence KC via oxidative stress mechanism(s). Thus, we investigated the possible association of polymorphisms rs61876744, rs138380, rs429358, rs7412, and *APOE* genotypes in KC patients of the Saudi population. This study included 98 KC cases and 167 controls. Polymorphisms rs6187644 and rs138380 were genotyped using TaqMan assays, and rs429358 and rs7412 were genotyped via Sanger sequencing. Although the allele frequency of rs61876744(T) in *PNPLA2* was a protective effect against KC (odds ratio (OR) = 0.64, 95% confidence interval (CI) = 0.44–0.93), the *p*-value (*p* = 0.020) was not significant for multiple testing correction (*p* = 0.05/4 = 0.015). However, rs6187644 genotype showed a modestly significant protective effect in the dominant model (OR = 0.53, 95% CI = 0.32–0.88, *p* = 0.013). Polymorphisms rs138380, rs429358, and rs7412 showed no significant allelic or genotype association with KC. However, the ε2-carriers (ε2/ε2 and ε2/ε3 genotypes) exhibited a greater than 5-fold increased risk of KC, albeit non-significantly (*p* = 0.055). Regression analysis showed no significant effect of age, gender, and the four polymorphisms on KC. Our results suggest that polymorphism rs6187644 in *PNPLA2* might be associated with KC in the Middle Eastern Arabs of Saudi origin but warrant a large-scale association analysis at this locus.

## 1. Introduction

Keratoconus (KC) is a degenerative disorder associated with the dysregulation of cellular and extracellular matrix (ECM) function, leading to progressive thinning and bulging of the cornea and loss of vision [1]. The prevalence of KC varies globally, ranging from 0.3% to 2.3% per 100,000 individuals [2,3]. KC can manifest early in life and progress asymmetrically, affecting each eye differently [4]. The therapeutic interventions of KC depend on the clinical stage of the disease. They may vary from contact lenses and corneal collagen UV-cross linking at early stages to corneal transplantation in progressive KC [4]. Therefore, it is essential to identify specific biomarkers that can facilitate early diagnosis. Consanguinity, family history, eye rubbing, contact lens wear, and allergy are some of the associated risk factors of KC [5,6,7]. Inflammation, oxidative stress, and ECM abnormalities are potential pathways involved in KC pathogenesis [8]. However, the exact etiology of KC remains elusive and is believed to involve an interaction between genetic and environmental factors [9,10,11]. Familial clustering, a variable inheritance pattern, and increased prevalence among Asian and Middle Eastern populations suggest a strong genetic link in KC [2,7,12].

In an attempt to examine the genetic risk of KC, many investigators have identified single nucleotide polymorphism (SNP) in several genes using linkage, whole-exome, genome-wide association study (GWAS) or candidate gene approaches [9,11,13,14]. These include *DOCK9*, *LOX*, *MIRNA184*, *HGF*, *RAB3GAP1*, *RXRA–COL5A1*, *FOXO1*, *FNDC3B*, *DCN*, *MPDZ-NF1B*, and *ZNF469* among many others. As a result of genetic heterogeneity, identifying the causative genes or genetic marker(s) responsible for KC needs further research to elucidate the underlying genetic mechanisms.

In a recent GWAS, McComish et al. identified two SNPs, rs61876744 located in the intronic region of the *PNPLA2* (patatin-like phospholipase domain–containing 2) gene and rs138380 near the *CSNK1E* (casein kinase I isoform epsilon) locus reaching a genome-wide significance for association with KC in populations of European ancestry [15]. *PNPLA2* locus is hypothesized to implicate apoptotic pathways in KC pathogenesis via *AP006621* gene, and SNP rs61876744 was associated with overexpression of *AP006621* (antisense RNA transcript *AP006621*) that might destabilize corneal structures and increase the risk of KC [15]. The protein-encoding *CSNK1E* has been implicated in controlling cytoplasmic and nuclear processes, including DNA replication and repair [16]. Variants in this gene have been associated with developmental and epileptic encephalopathy 1 [17]. However, little is known about this gene or polymorphism rs138380 plausible contribution to KC pathogenesis. It would thus be interesting to explore the possible association of these newly identified variants in a non-European KC cohort of Saudi origin.

Genetic polymorphisms in apolipoprotein E (*APOE*) have been implicated in various systemic diseases, age-related macular degeneration (AMD), glaucoma, and other neurodegenerative disorders [18]. Rs429358 and rs7412 are the two most commonly investigated polymorphisms in this gene [19]. These polymorphisms at codons 112 (rs429358; T>C) and 158 (rs7412; C>T) result in a Cys/Arg interchange, giving rise to three major allelic *APOE* variants ε2, ε3, and ε4 encoding Cys/Cys, Cys/Arg, and Arg/Arg, respectively [19]. APOE plays a crucial role in lipid homeostasis, maintaining and repairing neuronal cell membranes, coagulation, immunoresponse, and oxidative stress [20,21,22]. APOE knockout mice on a high-fat diet exhibit chronic inflammation, increased oxidative stress, impaired wound healing, and altered ECM remodeling [23]—processes that are all relevant to KC biology. APOE was reported to be the most significantly decreased protein in the KC corneal stroma [24]. In addition, the *APOE* variants have been reported to affect APOE levels [25,26]. It might thus be plausible that these variants may be associated with KC.

The underlying contribution of genes and genetic polymorphism(s) involved in KC in Middle Eastern Arabs remains unclear. The present study aimed to evaluate the possible association of rs61876744, rs138380, rs429358, rs7412, and APOE genotypes in KC patients of Saudi origin. To our knowledge, these SNPs have not been investigated in KC patients of Arab ethnicity.

## 2. Materials and Methods

### 2.1. Stduy Design and Cohort

A retrospective case–control genetic association study was performed adhering to the principles of the Declaration of Helsinki, where all the participants gave written informed consent. The ethical approval was obtained from the College of Medicine Institutional Review Board Committee, King Saud University, Riyadh, Saudi Arabia. Patients (n = 98), selected from the anterior segment clinic at King Abdulaziz University Hospital in Riyadh, were diagnosed with KC based on specific clinical criteria as described previously [27]—a Schimpff-flow-based elevation map showing posterior corneal elevation within the central 5 mm ≥ +20 μm, an inferior–superior dioptric asymmetry value > 1.2 diopters (D), and the steepest keratometry > 47D. All the participants were unrelated. Secondary cases of KC due to trauma, surgery, Ehlers–Danlos syndrome, osteogenesis imperfecta, and pellucid marginal degeneration, and patients with post-laser-assisted in situ keratomileusis (LASIK) ectasia were excluded. Healthy control participants (n = 167) of Saudi nationality with no ocular disease or history of ophthalmic surgery, clear cornea on examination, and normal Schimpff-flow-based elevation map were recruited from the general ophthalmology clinic [27]. Participants refusing to enroll in this study were excluded.

### 2.2. DNA Preparation

Blood samples drawn into EDTA tubes were used for DNA preparation. DNA extraction was performed using the QIAamp DNA Mini Kit (Cat. No. 51306, Qiagen, Hilden, Germany) following the specific protocols provided by the manufacturer. The DNA aliquots were stored at −80 °C until further use.

### 2.3. Genotyping of rs138380 (G>A) and rs61876744 (T>C)

Genotyping of rs138380 (G>A) near *CSNK1E* and rs61876744 (T>C) in *PNPLA2* was conducted using commercially available custom-designed TaqMan^®^ genotyping assay mix (Cat. No.: 4331349; Applied Biosystems Inc., Foster City, CA, USA) on ABI-7500 real-time PCR (Applied Biosystems). Real-time PCR amplification was conducted under recommended conditions in a 25 μL mix of 1X TaqMan^®^ genotyping master mix (Cat. No.: 4371355; Applied Biosystems), 1X SNP genotyping assay mix, and 20 ng DNA. Two negative controls (without DNA) were included in each 96-well plate. An in-built 2-color allele discrimination software version 2.0.5 was used for genotype calling. Probes and primers used for genotyping are described in Table 1.

### 2.4. Genotyping of rs429358 (T>C) and rs7412 (C>T)

SNPs rs429358 (T>C) and rs7412 (C>T) in the *APOE* gene were genotyped using PCR-based Sanger sequencing. The PCR reaction consisted of 1X PCR buffer, 250 µM dNTP mix, 100 pmoles of each primer, 1.5 U Taq polymerase, 1X Q-solution (Cat. No. 203205, Qiagen), and 20 ng of DNA. After stringent optimization, the target region was amplified using primers described in Table 2 using the optimum cycling conditions. Following confirmation of the amplified DNA (371 bp) via agarose gel electrophoresis and visualization in a UV gel documentation system (Bio-Rad, Hercules, CA, USA), the PCR products were purified using a QIAquick PCR Purification Kit (Cat. No. 28106, Qiagen) before being subjected to sequencing reactions. Sequencing was performed in both forward and reverse direction using M13 primers (Table 2) and BigDye Terminator V3.1 Cycle Sequencing kit (Applied Biosystems, Foster City, CA, USA). Samples were electrophoresed on the ABI 3730 genetic analyzer sequencer (Applied Biosystems) after removal of unincorporated dye terminators using DyeEx 96 Kit (Cat. No. 63183, Qiagen). The sequencing data was analyzed using CLC Sequence Viewer 6.0 (Qiagen, Hilden, Germany) to determine the nucleotide variations and *APOE* genotypes.

### 2.5. Statistics

The continuous variable was analyzed using the Mann–Whitney U-test after normality testing was conducted using Kolmogorov–Smirnov test. The deviation from Hardy–Weinberg equilibrium (HWE) and the categorical variable were examined using Chi-square and Fisher’s exact tests, where applicable. In addition, the frequency of genotypes was also compared using the Cochran–Armitage test for trend assuming additive model. Binary logistic regression analysis assessed the effects of multiple risk factors (age, sex, and genotypes) on KC. All the analysis was conducted using SPSS version 25 (IBM Inc., Chicago, IL, USA) and SNPStats online software version 1.0 (https://www.snpstats.net/start.htm accessed on 2 August 2023). Power analysis was performed using the PS program (version 3.1.2). A *p* < 0.05 was considered statistically significant. A Bonferroni’s correction *p*-value for multiple testing (*p* = 0.05/4 = 0.015) was applied where applicable.

## 3. Results

### 3.1. Demographic of Study Cohort

A total number of 265 subjects consisting of 98 KC and 167 controls were genotyped in this study. KC patients included sporadic (n = 67) and familial (n = 31) cases. Table 3 shows the age and gender distribution in cases and controls. The KC patients with a mean age of 25.8 (±7.3) ranging from 12 to 50 years were significantly younger (*p* < 0.001) than the controls with a mean age of 60.1 (±8.1) ranging from 35 to 78 years. Among the keratoconus patients, there were 55 males and 43 females compared to 88 males and 79 females in controls. The gender distribution was non-significant (*p* = 0.589).

### 3.2. Allelic Association Analysis

Table 4 represents the genomic location and minor allele frequency of the polymorphisms investigated in KC. SNPs rs138380, rs429358, and rs7412 were in HWE in the cases and controls, while SNP rs61876744 showed a slight deviation (*p* = 0.041) in KC cases. The frequency of rs6187674 (T) allele in *PNPLA2* was lower in KC (0.33) than in controls (0.43) and exhibited a significant protective effect against KC (OR = 0.64, 95% CI = 0.44–0.93, *p* = 0.020) but did not survive Bonferroni’s correction for multiple testing. There was no significant difference in allele frequencies of other SNPs between cases and controls.

### 3.3. Genotype Association Analysis

Genotype association analysis of polymorphisms in *PNPLA2* and near *CNSK1E* locus is represented in Table 5. Rs138380 near *CNSK1E* showed no association with KC in additive, dominant, and recessive genetic models. In contrast, rs61876744 in *PNPLA2* showed a modestly significant protective effect in the dominant model (OR = 0.53, 95% CI = 0.32–0.88, *p* = 0.013). Although the heterozygous T/C genotype of rs61876744 was also protective (OR = 0.53, 95% CI = 0.31–0.92) in the additive model, the *p*-value (*p* = 0.024) was insignificant for multiple testing. Likewise, the Cochran Armitage trend test assuming an additive model revealed 0.64-fold protection against KC but was insignificant for multiple corrections (*p* = 0.020).

The genotype distribution and analysis of polymorphisms rs429358 and rs7412 in the *APOE* gene is represented in Table 6. Overall, no significant genotype association of these polymorphisms with KC existed in any of the tested genetic models. The frequency of rs429358 C/C homozygous genotype was higher (3.1%) in cases than in controls (0%); likewise, the frequency of rs7412 C/T heterozygous genotype was 6.1% in KC compared to 4.2% in controls showing a 1.48-fold increased risk of KC. However, none of these differences were found to be statistically significant.

**Figure 1:**
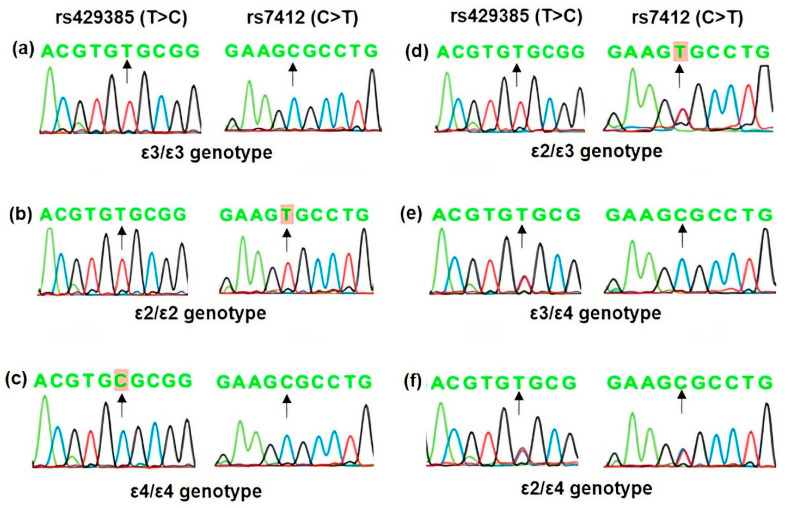
Representative DNA sequence chromatograms of *APOE* genotypes based on rs429358 (T>C) and rs7412 (C>T) polymorphisms. Arrows indicate the position of the nucleotide change and the homozygous nucleotide variant observed is highlighted. The nucleotide at the variant position in—(**a**) ε3/ε3 genotype is T/T and C/C, (**b**) ε2/ε2 genotype is T/T and T/T, (**c**) ε4/ε4 genotype is C/C and C/C at rs429358 and rs7412 polymorphisms, respectively, as indicated by arrows. Accordingly, the heterozygous—(**d**) ε2/ε3 genotype has T/T and C/T, (**e**) the ε3/ε4 genotype has T/C and C/C, and (**f**) ε2/ε4 has T/C and C/T at rs429358 and rs7412 polymorphisms, respectively, as shown by arrows.

The association analysis of the two *APOE* polymorphisms according to *APOE* alleles and genotypes is shown in Table 7. The allelic distribution was found in the order of ε3> ε4 > ε2 in both cases and controls. However, the distribution was non-significant. Likewise, ε3/ε3 was the most common genotype in both patients and controls, and the overall distribution of different types of apoε genotypes was statistically significant (Pearson Chi-Square = 16.54, df = 5, *p* = 0.0005). The ε2/ε3 genotype showed a significant association with KC (*p* = 0.012). However, a further analysis of *APOE* genotypes according to carrier status (Table 7) showed that the ε2-carriers (ε2/ε2 and ε2/ε3 genotypes) exhibited more than 5-fold increased risk of KC but were not statistically significant (*p* = 0.055). Representative sequence chromatograms of *APOE* genotypes based on rs429358 (T>C) and rs7412 (C>T) polymorphisms are shown in Figure 1.

### 3.4. Regression Analysis

Binary logistic regression analysis showed no significant impact of age (*p* = 0.079), sex (*p* = 0.077), rs138380 (*p* = 0.292), rs61876744 (*p* = 0.574), rs429358 (*p* = 0.232), and rs7412 (*p* = 0.982) polymorphisms on KC outcome (Table 8).

### 3.5. Power of This Study

Based on the allele frequencies observed in our cohort, this study had a power of 0.96, 0.97, and 0.72 per allele to detect a significant association between KC and SNPs rs138380, rs61876744, and rs429358, respectively, at an α level of 0.05 and an odds risk of 2.0. But, it was sufficiently underpowered (0.37) to detect any significant association for poly-morphisms rs7412.

## 4. Discussion

Keratoconus is a leading cause of visual morbidity in adolescents and young adults, with genetic, environmental, and behavioral traits contributing to the risk of KC [3]. Although a higher incidence of KC is reported in the Middle Eastern population compared to the Europeans [3], the genetic predisposition of KC in the Middle Eastern Arabs is predominantly unknown. It is thus essential to identify the genetic factors associated with KC in this population. This study examined the association of two newly identified polymorphisms, rs61876744 in *PNPLA2* and rs138380 near the *CSNK1E* locus [15], and the two commonly investigated variants in the *APOE* gene, namely the rs429358 and rs7412 [19], in KC patients of Saudi origin. We report no association of rs138380, rs429358, and rs7412 but a modest association of rs61876744 in the *PNPLA2* gene in our KC cohort.

McComish and colleagues [15] reported an association between two novel loci and KC at a genome-wide significance level in the Australian population using a genome-wide approach. These included rs61876744 in *PNPLA2* on chromosome 11 (*p* = 7.46 × 10^−9^) and rs138380 located 2.2 kb upstream of *CSNK1E* on chromosome 22 (6.35 × 10^−12^). Unlike the rs138380 near *CSNK1E*, the novel locus in *PNPLA2* was reported to remain significant in the replication analysis of the American, Irish, and Australian cohorts (*p* = 2.45 × 10^−8^).

According to the 1000Genomes NCBI database, the rs138380 (G) allele frequency is 0.47 in Europeans, 0.23 in Africans, 0.37 in Americans, and 0.28 in South Asians. The G allele frequency was reported to be 0.52 in controls and 0.38 in KC in the Australian cohort and was reported to be protective against KC. In comparison, in our study, the G allele frequency was 0.50 in controls and 0.52 in KC. However, we could not replicate an association of rs138380 near *CSNK1E* in our Saudi cohort. Likewise, in the GWA study by McComish et al. [15], the association of polymorphism rs138380 in KC was not replicated in the US, Ireland, and Australian replication cohort, suggesting that this variant may not have a significant role in KC. However, the significance of other polymorphisms near this locus cannot be ruled out. There are no other published studies of this polymorphism in different ethnicities in KC.

The rs61876744 (T) allele in *PNPLA2* has a frequency of 0.42 in Europeans, 0.30 in Americans, 0.35 in Africans, and 0.60 in South Asians, highlighting the existence of ethnic variations of this polymorphism (1000Genomes NCBI database). The rs61876744 (T) allele was reported to be protective (OR = 0.59) in the Australian, American, and Irish cohorts with the T allele frequency of 0.34 in KC and 0.44 in controls [15]. A similar protective effect of rs61876744 (T) allele (OR = 0.64) was observed in our study, having an allele frequency of 0.33 and 0.43 in KC and controls, respectively. Likewise, we observed a significant protective effect of rs61876744 genotypes in the dominant model (*p* = 0.013). However, the effect of this polymorphism was not independent of age, gender, and other SNPs included in this study, as observed in logistic regression analysis. Notably, the controls in our cohort were older than KC. Thus, the plausible presence of gene–gene and gene–environment interactions or the role of other causal variants that might be in linkage with this rs61876744 cannot be ruled out and needs further investigation.

PNPLA2 is a key enzyme catalyzing the first step of triglyceride hydrolysis in adipose and non-adipose lipid droplets [28]. PNPLA2 has been demonstrated to function as a retinyl ester hydrolase in the retinal pigment epithelium and an essential component of the visual cycle [29] and is suggested to be a potential therapeutic target in treating AMD [30]. The *PNPLA2* gene is highly expressed in all eye tissues, including the cornea [15], and is differentially expressed in the corneal epithelium in patients with KC and myopia [15,31]. Using the Genotype-Tissue Expression (GTex) data, McComish et al. identified an expression quantitative trait locus (eQTL) for an antisense RNA transcript AP006621 with rs61876744 in which the C allele was associated with increased levels of AP006621 transcript in the sun-exposed skin [15]. The authors hypothesized that the overexpression of AP006621 might destabilize corneal structures and the presence of rs61876744 (T) allele may reduce AP006621 expression and thereby decrease the risk of KC [15], thus exhibiting a protective effect. However, the exact pathogenetic mechanism(s) by which *PNPLA2* might be involved in KC remains to be investigated.

The individual analysis of polymorphisms rs429358 and rs7412 in the *APOE* gene showed no significant allelic or genotype association with KC. The allele frequencies of these SNPs observed in our cohort were consistent with the global frequency reported for these SNPs across different ethnicities (1000Genomes NCBI database). Further analysis of these polymorphisms was performed according to *APOE* alleles (ε2, ε3, and ε4), and the resulting six genotypes (ε2/2, ε2/3, ε2/4, ε3/3, ε3/4, and ε4/4) [19]. Globally, the frequency of allelic variation in the *APOE* locus lies in the range of 60–90% for ε3, 0 to 20% for ε2, and 10–20% for ε4 alleles, respectively [32]. Similarly, ε3 was the most predominant allele, followed by ε4 and ε2 in both our study groups, but showed no significant association with KC. Although the ε2/ε3 genotype was significantly associated with an increased risk of KC, the significance was lost when ε2 carriers were analyzed. There are no published reports on the association of *APOE* genotypes in KC. Several investigators have reported ε4/ε4 to be associated with an increased risk of CVD, diabetes, and Alzheimer’s [33,34,35]. In contrast, ε2/ε2 has been reported to be associated with increased risk in AMD [36,37]. It has been demonstrated that the APOE isoforms exhibit differences in net charge and that their cell-specific binding properties and function may vary depending on the target cell type [38,39]. These differences may explain the variable effects of APOE2 or E4 isoforms.

Beyond the role of APOE in lipid transport, apoE deficiency has been demonstrated to promote increased oxidative stress in an APOE isoform-dependent manner [40,41,42]. Oxidative stress plays a critical role in the pathophysiology of KC, and the cornea is particularly susceptible to oxidative damage [2,43]. A high ROS production, decreased antioxidant status, and increased mitochondrial DNA damage can eventually lead to ECM dysfunction in the stroma, triggering stromal thinning [43]. Similar to the reported studies in AMD [36,37], a significantly five-fold increased risk of KC was observed in our patients with ε2-carriers, but the effect was non-significant. The lack of significance in our cohort may be related to fewer numbers in each genotype group and needs further evaluation in a much larger cohort.

This study has certain limitations, and the results thus need careful interpretation. The number of samples analyzed in this study is relatively small, with much smaller numbers in different subgroup analyses, which may affect the power of this study. Our study had sufficient power to detect an odds of 2.0 for all the polymorphisms investigated in this study, except for rs7412. However, a larger sample number should be examined to detect an odds of ≤1.5, as commonly reported in genetic association studies to establish a strong association. This study provides no functional or mechanistic evidence. Since ours is a tertiary care center, there could be a possible referral or selection bias that may not truly represent the general Saudi population. Also, considering the significant role of epistatic/gene–environment interactions in KC further accentuates the need to confirm these results in well-designed, sizeable, population-based samples.

In conclusion, our study suggests that polymorphism rs61876744 in the *PNPLA2* gene is associated with KC in Middle Eastern Arabs of Saudi origin. However, the results warrant further replication in a large-scale association analysis with cohorts from multiple centers, possibly with age- and gender-matched controls, to confirm the risk rs61876744/*PNPLA2* locus may confer in the development or progression of KC.

## Figures and Tables

**Table 1:** Primer and probe sequences used in TaqMan^®^ assays for genotyping rs138380 and rs61876744 polymorphisms.

rs138380	Primers and Probe Sequences (5′–3′)
**Forward Primer**	GGGAAACAATCAAATATTTTGACAAATAATCGT
**Reverse Primer**	CTCAGAAAATAATTCAGTAGCAACAAGGT
**Probe Reporter dye [VIC/FAM]**	CCAGGAATC[T/C]CCTTGTT
**rs61876744**	
**Forward Primer**	TGAACTTTGTCCTGGGAGGGA
**Reverse Primer**	GGCTGTTCCCAATAATAGCTCTAGT
**Probe Reporter dye [VIC/FAM]**	CAGAAGTGAACC[T/C]CTCAGG

**Table 2:** PCR and sequencing primers used for *APOE* genotyping.

PCR Primers	Primer Sequences (5′–3′)
**APOE-Forward**	**TGTAAAACGACGGCCAGT**GACCATGAAGGAGTTGAAGGCCTAC
**APOE-Reverse**	**CAGGAAACAGCTATGACC**GATGGCGCTGAGGCCGCGCT
**Sequencing Primers**	
**M13-Forward**	TGTAAAACGACGGCCAGT
**M13-Reverse**	CAGGAAACAGCTATGACC

Bold and underlined sequences represent M13 sequence.

**Table 3:** Demographic data for each group.

Type	n	Mean Age, Years (SD)	% Male
Keratoconus	98	25.8 (7.3)	56
Controls	167	60.1 (8.1)	52

**Table 4:** Polymorphism details and allele frequencies observed in controls and Keratoconus cases.

SNP ID	Gene/ Locus	Chromosome	Position *	Minor Allele	Minor Allele Frequency		Odds Ratio (95% Confidence Interval)	*p*-Value
					Controls	Cases		
rs138380	*CSNK1E*	22q13.1	38400624	G	0.50	0.52	1.06 (0.74–1.51)	0.729
rs61876744	*PNPLA2*	11p15.5	820754	T	0.43	0.33	0.64 (0.44–0.93)	0.020
rs429358	*APOE*	19q13.32	44908683	C	0.09	0.10	1.05 (0.57–1.91)	0.887
rs7412	*APOE*	19q13.32	44908821	T	0.03	0.03	1.14 (0.40–3.25)	0.806

* Genomic base pair position per GRCh38/hg38.

**Table 5:** Association analysis of polymorphisms near *CNSK1E* locus (rs138380) and in *PNPLA2* (rs61876744) in Keratoconus.

SNP ID	Genetic Model	Genotype	Control n (%)	Cases n (%)	Odds Ratio (95% Confidence Interval)	*p*-Value
**rs138380**	Additive	A/A	43 (25.8)	27 (27.6)	1.00	-
		G/A	81 (48.5)	41 (41.8)	0.80 (0.437–0.148)	0.488
		G/G	43 (25.8)	30 (30.6)	1.11 (0.56–2.17)	0.764
	Dominant	A/A	43 (25.8)	27 (27.6)	1.00	
		G/G-G/A	124 (74.2)	71 (72.5)	0.91 (0.52–1.60)	0.751
	Recessive	G/A-A/A	124 (74.2	68 (69.4)	1.00	
		G/G	43 (25.8)	30 (30.6)	1.27 (0.73–2.20)	0.392
	CA trend *		167/167	101/95	1.06 (0.74–1.51)	0.729
**rs61876744**	Additive	C/C	56 (33.5)	48 (49)	1.00	-
		T/C	79 (47.3)	36 (36.7)	0.53 (0.31–0.92)	0.024
		T/T	32 (19.2)	14 (14.3)	0.51 (0.24–1.07)	0.071
	Dominant	C/C	56 (33.5)	48 (49)	1.00	
		T/C-T/T	111 (66.5)	50 (51)	0.53 (0.32–0.88)	0.013
	Recessive	C/C-T/C	135 (80.8)	84 (85.7)	1.00	
		T/T	32 (19.2)	14 (14.3)	0.70 (0.35–1.39)	0.310
	CA trend *		143/191	64/132	0.64 (0.44–0.93)	0.020

* CA Cochran Armitage trend test assuming additive model.

**Table 6:** Association analysis of polymorphisms in *APOE* gene in Keratoconus.

SNP ID	Genetic Model	Genotype	Control n (%)	Cases n (%)	Odds Ratio (95% Confidence Interval)	*p*-Value
**rs429358**	Additive	T/T	136 (81.4)	82 (83.7)	1.00	-
		T/C	31 (18.6)	13 (13.3)	0.70 (0.34–1.41)	0.310
		C/C	0 (0)	3 (3.1)	NA (0.00–NA)	0.055 ^†^
	Dominant	T/T	136 (81.4)	82 (83.7)	1.00	
		T/C-C/C	31 (18.6)	16 (16.3)	0.86 (0.44–1.66)	0.640
	Recessive	T/T-T/C	167 (100)	95 (96.9)	1.00	
		C/C	0 (0)	3 (3.1)	NA (0.00–NA)	0.014
	CA trend *		31/303	19/177	1.05 (0.57–1.91)	0.887
**rs7412**	Additive	C/C	159 (95.2)	92 (93.8)	1.00	-
		C/T	7 (4.2)	6 (6.1)	1.48 (0.48–4.52)	0.560
		T/T	1 (0.6)	0 (0)	NA (0.00–NA)	1.00 ^†^
	Dominant	C/C	159 (95.2)	92 (89.8)	1.00	
		C/T-T/T	8 (4.8)	6 (10.2)	1.29 (0.43–3.85)	0.639
	Recessive	C/C-C/T	166 (99.4)	98 (100)	1.00	
		T/T	1 (0.6)	0 (0)	NA (0.00–NA)	0.340
	CA trend *		9/325	6/190	1.14 (0.39–3.25)	0.806

* CA Cochran Armitage trend test assuming additive model, NA not available, ^†^ Fisher exact test.

**Table 7:** Association analysis of *APOE* polymorphisms according to *APOE* alleles and genotypes.

*APOE*	Controls n (%)	Cases n (%)	Odds Ratio (95% Confidence Interval)	*p*-Value
**Alleles**				
ε3	294 (88.0)	171 (87.2)	1.00	-
ε2	9 (2.7)	6 (3.1)	1.14 (0.40–3.27)	0.806
ε4	31 (9.2)	19 (9.7)	1.08 (0.59–1.96)	0.862
**Genotypes**				
ε2/ε2	01 (0.6)	00 (0)	0.00 (0.00–NA)	0.770
ε2/ε3	01 (0.6)	06 (6.1)	10.5 (1.25–89.52)	0.012
ε2/ε4	06 (3.6)	00 (0)	0.00 (0.00–NA)	0.092
ε3/ε3	134 (80.2)	76 (77.5)	1.00	-
ε3/ε4	25 (15.0)	13 (13.3)	0.91 (0.44–1.89)	0.823
ε4/ε4	00 (0)	03 (3.1)	0.00 (0.00–NA)	0.095
**Carrier ^a^**				
ε3/ε3	134 (83.2)	76 (80.8)	1.00	-
ε*2 ^b^	02 (1.2)	06 (6.4)	5.28 (1.04–26.8)	0.055
ε*4 ^c^	25 (15.5)	16 (12.8)	1.12 (0.56–2.24)	0.730

^a^ ε2/ε4 were excluded from either ε*2 or ε*4 group, ^b^ includes ε2/ε2 and ε2/ε3, ^c^ includes ε4/ε4 and ε3/ε4. NA not available.

**Table 8:** Binary logistic regression analysis.

Group Variables	B	SE	Wald	*p*-Value
Age	−1.102	0.627	3.090	0.079
Sex	3.859	2.182	3.127	0.077
rs138380			2.462	0.292
G/A	9.775	6.553	2.225	0.136
G/G	5.520	4.783	1.332	0.248
rs61876744			1.111	0.574
C/T	−0.298	2.305	0.017	0.897
T/T	3.214	3.051	1.110	0.292
rs429358			2.262	0.323
T/C	−0.603	0.401	2.262	0.133
C/C	21.578	23,051.628	0.000	0.999
rs7412			0.036	0.982
C/T	1.473	7.727	0.036	0.849
T/T	10.171	27,454.935	0.000	1.000
Constant	35.883	20.081	3.193	0.074

## Data Availability

The data supporting the conclusions of this article are all presented within this report.
